# ENHANCING HOME-BASED CARE WITH ASSISTIVE ROBOTS: ACCEPTANCE AND PREFERENCES OF OLDER CHINESE

**DOI:** 10.1093/geroni/igae098.0349

**Published:** 2024-12-31

**Authors:** Yilin Wang, Hongxiu Chen, Xiuying Hu, Yan Qi

**Affiliations:** Innovation Center of Nursing Research and Nursing Key Laboratory of Sichuan Province, West China Hospital, Sichuan University, Chengdu, Sichuan, China (People’s Republic); Innovation Center of Nursing Research and Nursing Key Laboratory of Sichuan Province, chengdu, Sichuan, China (People’s Republic); Innovation Center of Nursing Research and Nursing Key Laboratory of Sichuan Province, West China Hospital, Sichuan University, Chengdu, Sichuan, China (People’s Republic); China Rehabilitation Research Center, beijing, Beijing, China (People’s Republic)

## Abstract

This study aimed to assess the acceptance and preferences of the older Chinese towards assistive robots, focusing on their potential to aid in mobility, provide emergency response, and monitor daily activities. A structured survey questionnaire was distributed to evaluate their difficulties in daily activities, the use and expenditure on current eldercare assistive devices, and their interest in and concerns regarding assistive robots. Among 109 who responded, 85 were valid. More than half were over age 75, including those aged between 75 and 84 years old (37.7%) and those aged 85 and above (30.6%). Participants reported a strong interest in assistive robots with mobility aid, emergency response, and daily activity monitoring. As most of the care is provided at home (96.5%), significant difficulties were reported in activities such as stair navigation (67.1%), outdoor transportation (63.5%), bathing (55.3%), and toileting (47.1%). Canes (69.4%) and wheelchairs (60%) were the most used eldercare assistive devices, with expenditures mostly below 5,000 Chinese RMB (50.7%). Safety (81.2%) and ease of use (61.2%) were the primary considerations. Approximately 60% of respondents prioritized mobility assistance, while around 40% valued the ability of robots to provide companionship. Despite the interest in assistive robots, concerns over cost and the need for user-friendly interfaces were highlighted as potential barriers to adoption. The study underscores the potential of assistive robots in elder care, particularly in enhancing mobility and ensuring safety. To facilitate wider adoption, it is crucial to ensure that these technologies are affordable and accessible.

